# Morphotype broadening of the grapevine (*Vitis vinifera* L.) from Oxus civilization 4000 BP, Central Asia

**DOI:** 10.1038/s41598-022-19644-0

**Published:** 2022-09-29

**Authors:** Guanhan Chen, Xinying Zhou, Mutalibjon Khasannov, Robert N. Spengler, Jian Ma, Tukhtash Annaev, Nasibillo Kambarov, Farhod Maksudov, Jianxin Wang, Akhmadali Askarov, Xiaoqiang Li

**Affiliations:** 1 Key Laboratory of Vertebrate Evolution and Human Origins of Chinese Academy of Sciences, Institute of Vertebrate Paleontology and Paleoanthropology, Chinese Academy of Sciences, Beijing, 100044 China; 2 CAS Center for Excellence in Life and Paleoenvironment, Beijing, 100044 China; 3 University of Chinese Academy of Sciences, Beijing, 100049 China; 4 Institute of Archaeology, Uzbek Academy of Sciences, Samarkand, 140151 Uzbekistan; 5 Department of Archaeology, Max Planck Institute for the Science of Human History, 07745 Jena, Germany; 6 College of Cultural Heritage, Northwest University, Xi’an, 710069 China; 7 History Department, Termez State University, Termez, 190100 Uzbekistan

**Keywords:** Archaeology, Cultural evolution

## Abstract

The region of Transoxiana underwent an early agricultural-demographic transition leading to the earliest proto-urban centers in Central Asia. The agronomic details of this cultural shift are still poorly studied, especially regarding the role that long-generation perennials, such as grapes, played in the cultivation system. In this paper, we present directly dated remains of grape pips from the early urban centers of Sapalli and Djarkutan, in south Uzbekistan. We also present linear morphometric data, which illustrate a considerable range of variation under cultivation that we divide into four distinct morphotypes according to pip shape. While some of the pips in these two assemblages morphologically fall within the range of wild forms, others more closely resemble modern domesticated populations. Most of the specimens measure along a gradient between the two poles, showing a mixed combination of domesticated and wild features. We also point out that the seeds recovered from the Djarkutan temple were, on average, larger and contained more affinity towards domesticated forms than those from domestic contexts. The potential preference of morphotypes seems to suggest that there were recognized different varieties that local cultivators might aware and possibly propagating asexually.

## Introduction

The Greater Khorasan Civilization^1^ is one of the least studied ancient centers of intensive agricultural development, urbanization, and imperial expansion. Stretching back to Possehl’s^2^ Middle Asian Interaction Spheres, it has been archaeologically clear that the early populations of southern Central Asia were intertwined into long-distance trade networks, which, among other cultural attributes, fostered an early globalization process in the agricultural arena^3–5^. Across the river ways and oases of southern Central Asia, a complex agricultural system gradually developed from 7000 to 5000 BP^6–8^. By the early 5000 BP, large-scale irrigated agriculture projects were fostering early urbanization and the establishment of an elite class^9,10^. Increased localized human density and regional demographic growth presumably further increased the demand for crop diversity and appears to have promoted the emergence and development of arboriculture, which would, hypothetically, have been tied to increased sedentism, greater stability in land tenure, and more reliable political oversight of irrigation systems. Recently Fuller and Stevens^11^ resurrected Childe’s^12,13^ concepts, suggesting that there was a direct link within Eurasian cultural development between the rise of arboriculture and the expansion of urbanism. The earliest evidence for the cultivation of grapes (*Vitis vinifera*), and possibly apples (*Malus pumila*) and plums (*Prunus* cf. *insititia*) in Central Asia comes from this time period and region^14^. The cultivation of long-generation perennials requires different cultivation techniques from those used for annual crops, including grafting and cloning, and, in these arid regions, reliable irrigation systems^15,16^. The grape seeds that we present in this manuscript represent some of the best evidence for the beginnings of perennial cultivation in this understudied ancient agricultural center.

The burgeoning urban centers across the oases of the southern Kara Kum and river valleys of the Kopet Dag grew in scale and population size starting around 5500–5000 BP^17,18^. Archaeologists working in this region have emphasized the magnitude of exchange networks and globalization processes of this part of the world during this period^2,17,19–21^. The unique cultural repertoire that came to represent these clusterings of cities has been variably referred to in archaeological literature as the Oxus Civilization^22^, Bactria-Margiana Archaeological Complex (BMAC)^17,18,23^ and Greater Khorasan Civilization^1^. In many aspects, these peoples reached a demographic zenith around four millennia ago, when agricultural investment and craft production was heavily intensified^14,18^.

**Figure 1:**
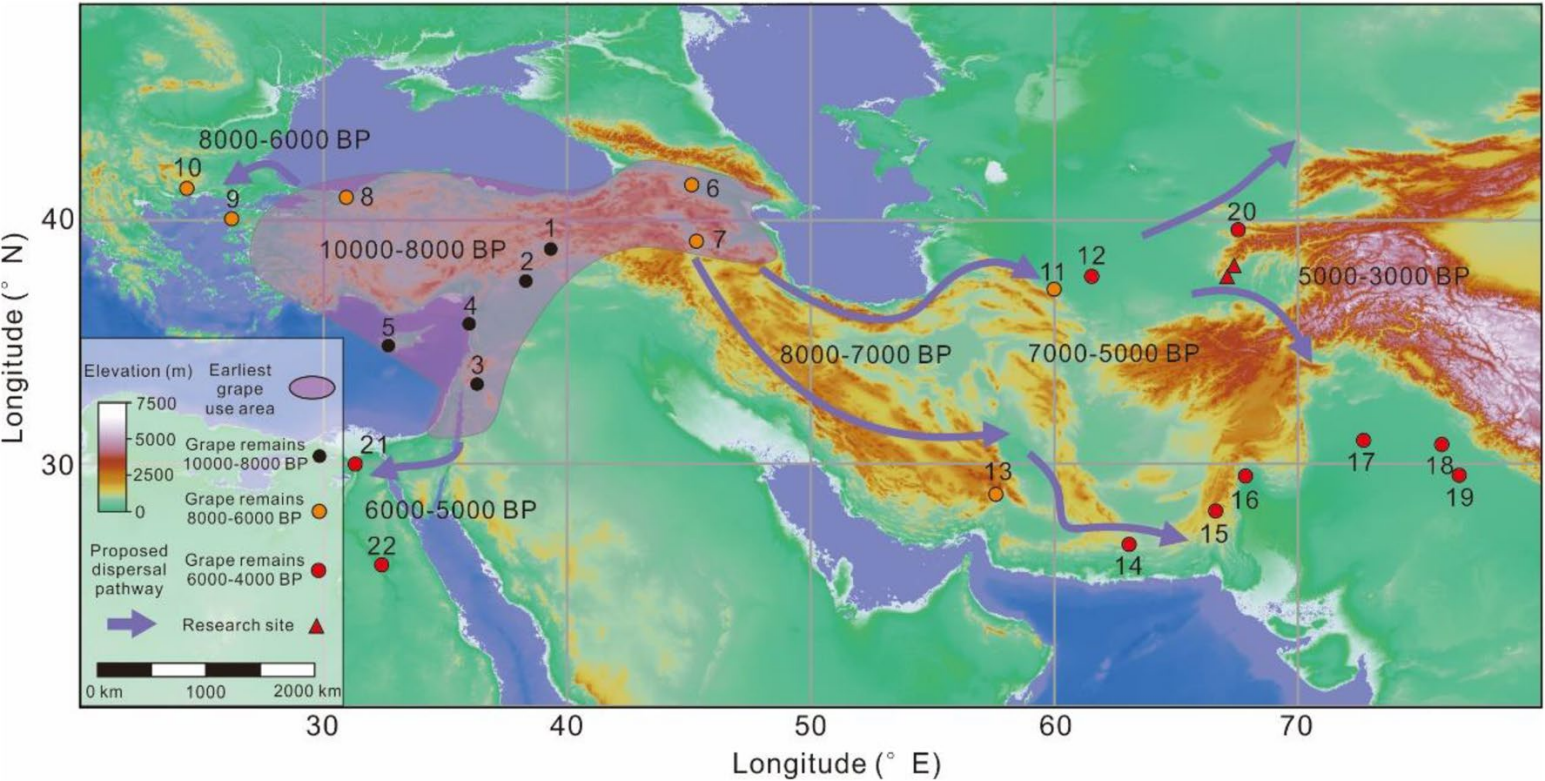
Site distribution of early grape use, evidence and possible diffusion paths. 1. Cayonu (10,800–10,300 BP); 2. Tell Halula (10,000–9100 BP); 3. Jericho (11,150–10,300 BP); 4. Tell Aswad II (10,200–9500 BP); 5. Prastio Mesorotsos (9800–9200 BP); 6. Khramis DidiGora (8000–7000 BP); 7. Aratashen (7840–7640 BP); 8. Ilipinar (8030–7800 BP); 9. Kumtepe (7000–5000 BP); 10. Dikili Tash (6000–3200 BP); 11. Monjukly Depe (7000–6500 BP); 12. Gonur Tepe (4200–3700 BP); 13. Konur Sandal South (4800–4200 BP); 14. Miri Qalat (6000–5500); 15. Sohr Damb (4700–4400 BP); 16. Mehrgarh (5000–4000 BP); 17. Harappa (5000–4500 BP); 18. Rohira (4900–4500 BP); 19. Balu (4500–3900 BP); 20. Sarazm (4600–4000 BP); 21. Tell Ibrahim Awad (5300–4900 BP); 22. El Abadiya 2 (5960–5640 BP). This maps were created using ArcGIS v10.6 (https://www.esri.com/), in-map labels were added in CorelDraw X8 v18.1.0.690 (https://www.coreldraw.com/). Reference in ESM Table S1.

It is well accepted that the domesticated grape evolved from wild *Vitis vinifera* ssp. *sylvestris* populations in parallel across the broad area of southeastern Europe and southwest Asia^24^. The domesticated grape gradually spread into Europe and East Asia and became one of the most important woody perennial crops in the world (Fig. 1). Several factors have complicated studies of the first evolution of domestication traits in the grape: (1) modern cultivar grapes tend to be asexually reproduced; (2) preferred lineages have been asexually propagated for centuries; and (3) the he main domestication trait was likely hermaphroditism, which is not archaeologically visible. Large-scale population genetics studies of modern grape accessions support a broad domestication center across southwest Asia and a weak domestication bottleneck resulting from continual crop-to-wild gene flow and millennia of vegetative propagation used to maintain distinct cultivars^25^. It still not clear why early farmer chose grapevine for domestication, Miller^26^ argued that the rapid and widespread adoption of grapes across Eurasia was a response to the human desire for sweetness, as opposed to alcohol. Whatever the driving cultural mechanisms, the grape tended to pioneer the way for the adoption of other long-generation perennial crops, as it was easily cultivated, readily propagated asexually (locking preferred traits in place and reducing the number of seasons until the first harvest), and relatively drought, heat, and frost (winter not spring frosts) tolerant. Once peoples across Eurasia mastered viticulture, the transition into other forms of arboriculture, notably of asexually propagated rosaceous species, was a logical next step, and the grape may have paved the way for the domestication of the apple and plum.

**Figure 2:**
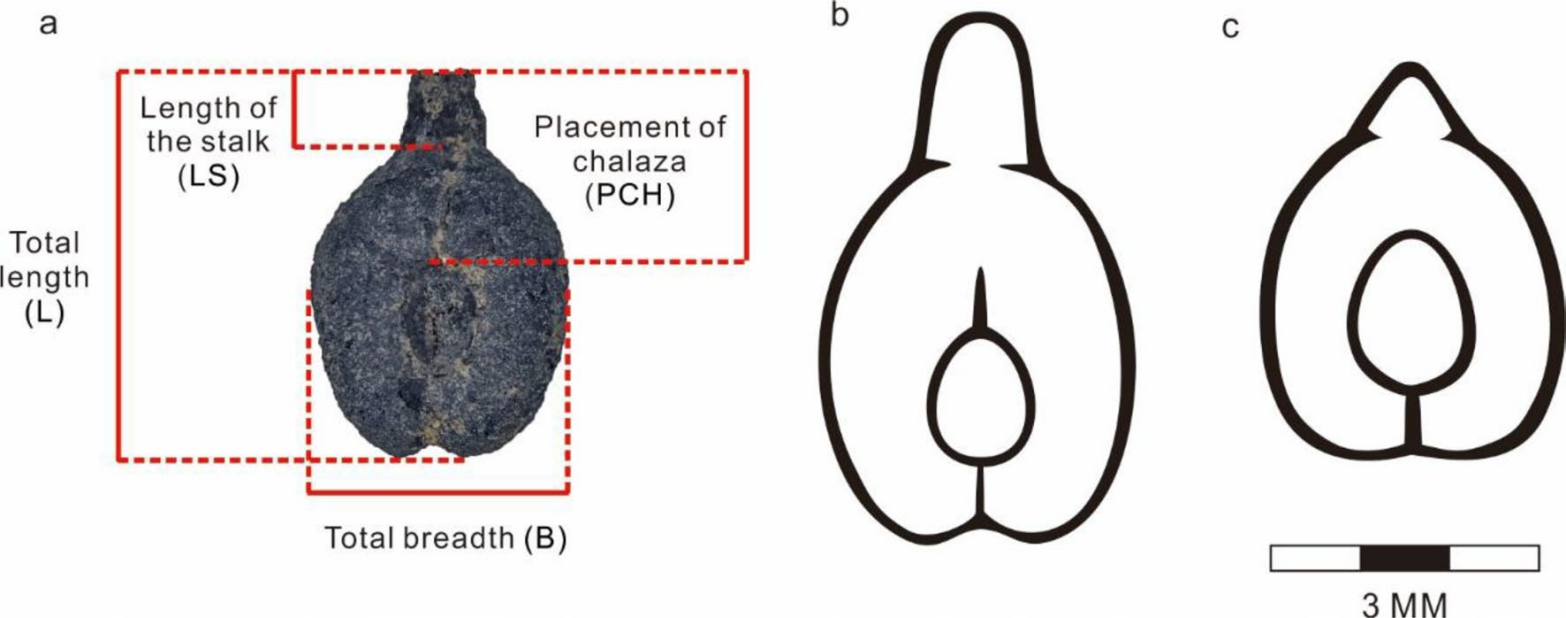
Dorsal view of an archeological specimen with indications of linear morphometric measurements (**a**) and morphological differences between modern cultivated grapes (**b**) and wild grapes (**c**).

The most significant evolutionary changes under cultivation within the grape include an increase in the mass of the pericarp and the size of the inflorescence, greater sugar content, and more regular yeilds^27^; Although, it remains unclear when humans began recognizing distinct forms and locking them in place as genets through cloning^28^. The main characteristics related to domestication do not preserve archaeologically. While aDNA and population genetics of historical landraces has made headway in answering many of these questions over the past decade^29–31^, directly dated evidence for trait evolution is still needed. Some studies have attempted to link historical and archaeological lineages genetically^29,32,33^, but most archaeobotanical plant remains are carbonized and therefore not suitable for genetic analysis. The use of image analysis techniques for multivariate comparisons and classification of modern and archaeological samples has been increasing in prominence (Fig. 2)^34–36^. Over the past few years, morphometric methods have been more routinely applied to the study of grain, legume, and fruit seeds, proving their effectiveness in assessing variation within ancient populations^35,37,38^. The method has not only proven effective among ancient Mediterranean grape populations^38,39^, but also for cherries (*Prunus avium*)^40^, dates (*Phoenix dactylifera*)^41^, and olives (*Olea europaea*)^42^. More recently, the integration of 3D seed scanning techniques, machine-learning software, and genetic tests are further proving the effectiveness of the morphometric approaches^43^. Despite the integration of new measurement methods, the effectiveness and reliability of linear morphometrics for the study of variation in grape seeds has been illustrated through repeated studies in the Mediterranean.

In this study, we examine two populations that represent some of the earliest cultivated grape assemblages in southern Central Asia, originating from the early urban sites of Sapalli (n = 21) and a temple at Djarkutan (n = 103) (Archaeological background in ESM 2). We selected only seeds that appeared well preserved to avoid deformation due to taphonomy or carbonization. The goal of this study is to better understand early horticultural activities, specifically whether distinct genets or clonal forms were recognized, preferred, and propagated by different groups of people during early urban development among the oasis civilizations of Central Asia.

### Dating of archaeobotanical samples

**Figure 3:**
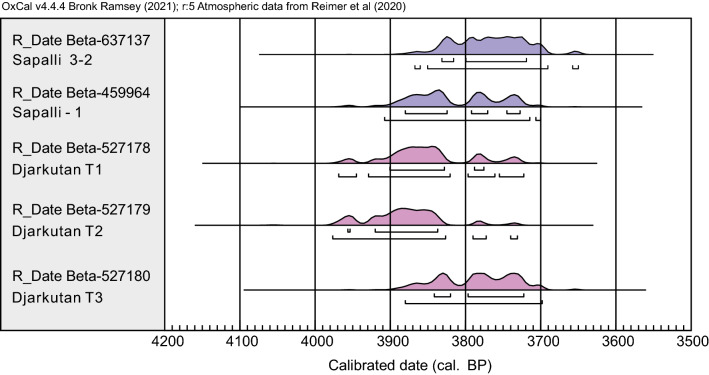
AMS^14^C dating results of Sapalli and Djarkutan temples.

Five seeds were selected from the Sapalli and Djarkutan assemblages (Fig. 3, Table S1) for radiocarbon analysis. Grape seeds were relatively rare in the Sapalli assemblage, so we selected 1 wheat seed and 1 uncharred grape fragment for AMS^14^C date. The results ranged from 4086 to 3650 cal. BP corresponding to the Late Bronze Age for this region; these findings are consistent with previous research at the site. From the temple at Djarkutan, a total of 3 specimens of carbonized grape seeds were selected for AMS^14^C dating. These seeds ranged in age from 3976–3702 cal. BP, falling roughly contemporaneously with Sapalli. The dating of these two sites both supports earlier dating systems that were based largely on material culture and also shows that the urban sites were roughly contemporaneously occupied. Some of the preserved grape pips from Sapalli were only partially carbonized, which appears to have been possible at this site due to the extreme aridity and partial mineralization. Other sites in Central Asia have produced desiccated plant material, and grapes that are prone to survival in a non-carbonized state^44^. Our dating campaign here has ensured that these are, indeed, ancient seeds and not modern intrusive specimens.

### Morphotyping the archaeological grape pips

**Figure 4:**
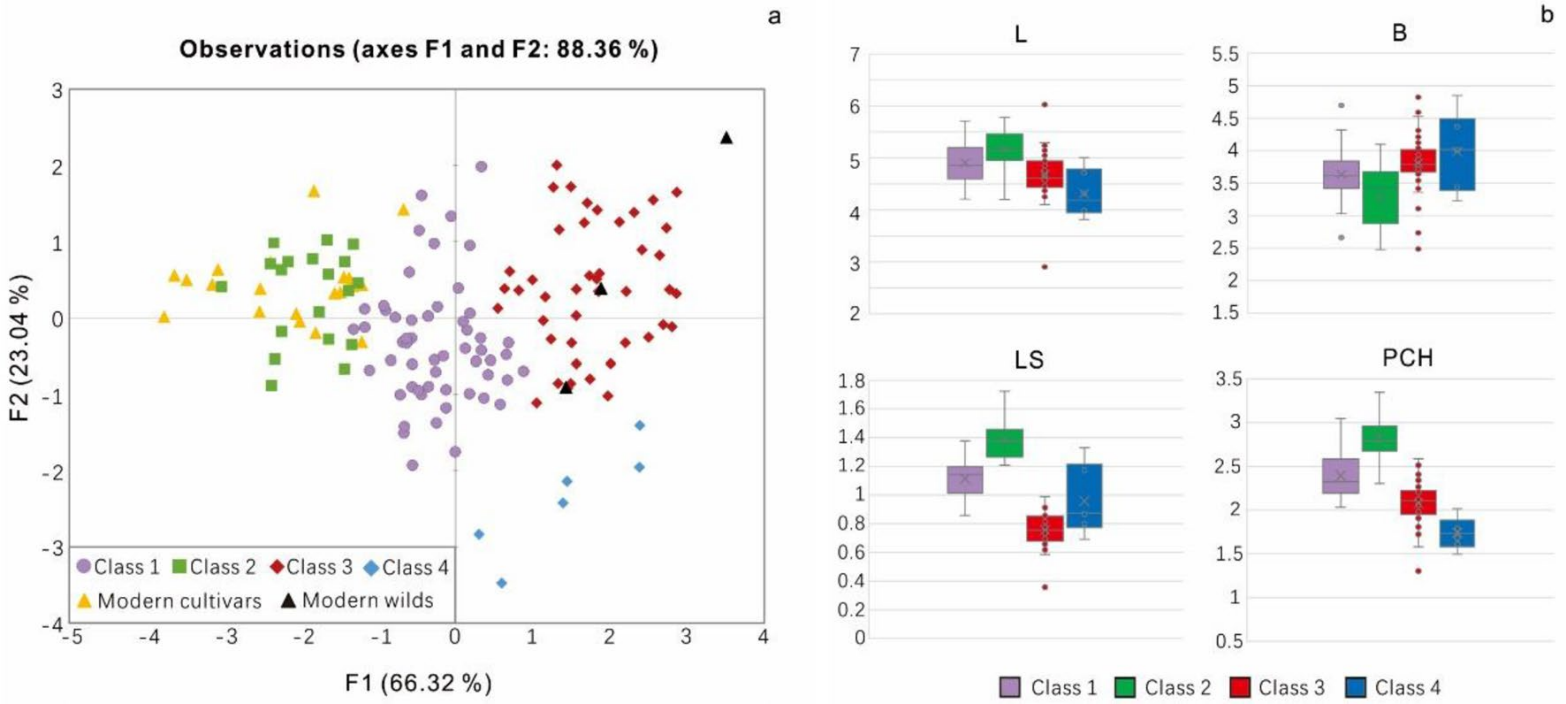
The results of PCA result of archeological grape pips from the Bronze Age in Central Asia and modern domesticated grape pips and parameter changes in each class. (**a**) PCA results based on centroid coordinates of PC1 and PC2 (Total variance 88.36%). (**b**) Mean and variance of L, B, LS, and PCH data in each class (PCA and LDA detail in EMS 3).

Building on the growing number of studies of grape pip morphometrics, we seek to add a new perspective, as most previous studies were conducted in the Mediterranean. While the scholars conducting many of these other studies attempted to link modern or historical landraces to archaeobotanical remains, we approach this practice cautiously. However, we did add an assemblage of modern wild and cultivar grape pips^37,45,46^, collected from western Europe and Mediterranean region for comparison. The results of PCA (Fig. 4a) show that some grape seeds found in the archaeological contexts share certain similarities with modern cultivars, but there were still obvious morphological differences between the populations. The first two principal components (PCs) of the PCA explained 88.36% of the total variance (Fig. 4a). Among them, PC1 (66.32% of variance) is, in particular, correlated with the variable of Log-Shape LS (R = 0.916), and negatively correlated with the Log-Shape B variable (R = -0.904), which illustrated a contrast between roundish pips with a short stalks and the rest of the assemblage. Some morphological observations have suggested that the progenitor of the domesticated grape, *V. sylvestivis*, has shorter pips and stalks^28,37^. Therefore, the differences in PC1 exhibited across different specimens could, hypothetically, represent differences in the stage of domestication; although, domestication in grapes, as with many other crops appears more as a mosaic of characteristics than a neat linear trajectory. Also, due to variation resulting from hybridization, thinking about a clear gradient towards more domesticated forms, as has been illustrated for annual grain crops, does not necessarily apply in this context. PC2 (23.04% of variance) is correlated with the position of the chalaza (Log-Shape PCH, with R = 0.837).

**Figure 5:**
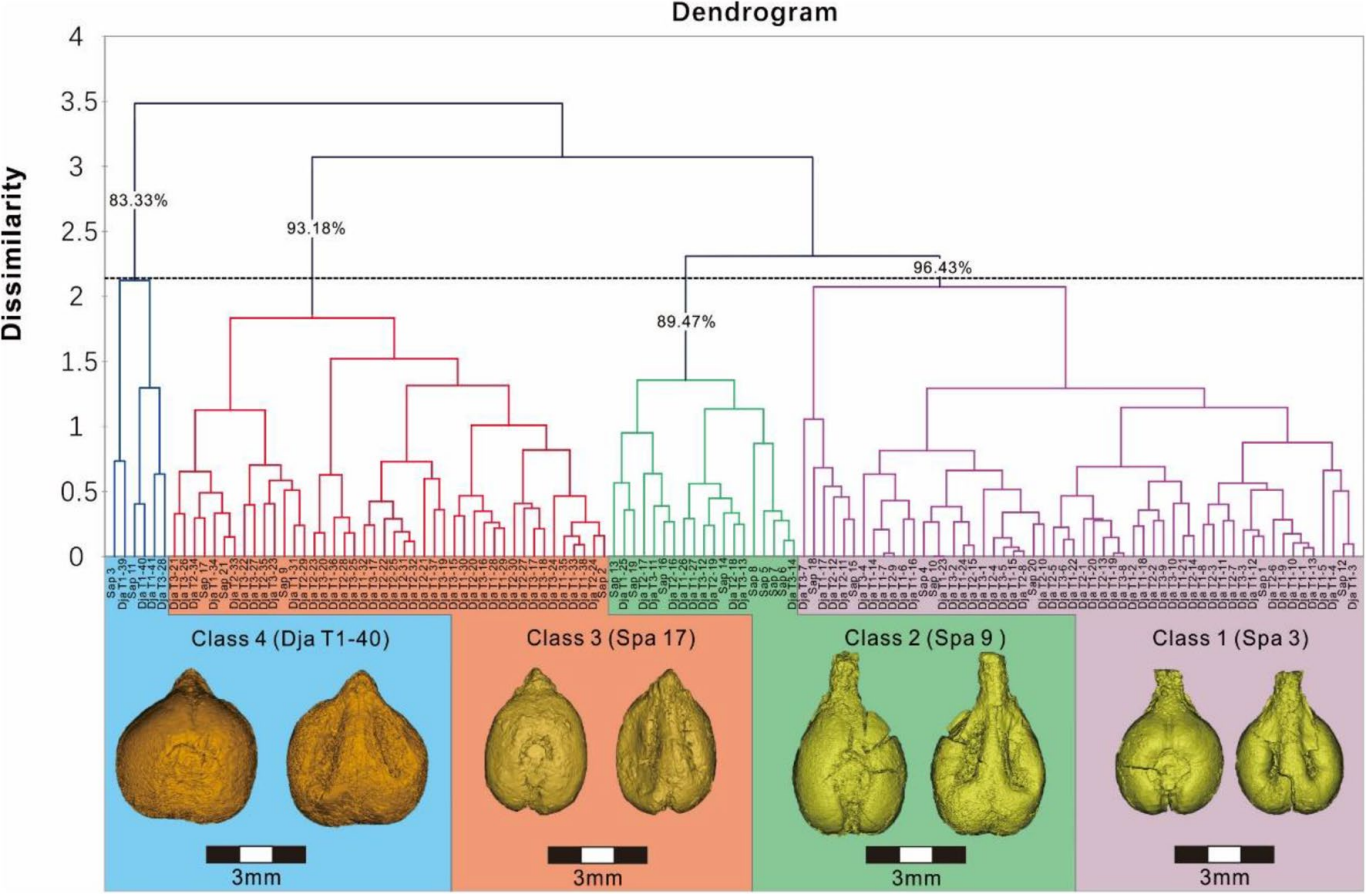
UPGMA cladogram of sub-groups identified at an arbitrary Euclidian distance of 2 (dotted line) with indication of the discrimination rate (%) calculated by LDA and typical archaeological sample 3D module of each class. Dja = Djarkutan; Spa = Sapalli; Purple line = Class 1; Green line = Class 2; Red line = Class 3; and Blue line = Class 4.

The UPGMA cladogram (Fig. 5) shows 4 main morphological classes of grape seeds in this study, which can be divided into two segments. The most abundant classes, 1–3, were all clustered in one segment, accounting for 95.2% of all seeds in this study, while independent class 4, which was rare, accounted for only 4.8% of the assemblage, represented the other segment. The statistics of L, B, LS, and PCH (Fig. 4b) show that class 4 has obvious morphology differences compared with the others, including short length (L), a comparatively wide breath (B), and short chalaza (PCH), some of these seeds are even wider than they are long. These differences are too extreme to account for simply through environmental factors and developmental plasticity. Within the portion that accounts for classes 1, 2, and 3, class 3 has the shortest stalk (LS) and its breath (B) is wider than that of classes 1 and 2. In this one landmark, class 3 seeds share a similarity to those of class 4. The differences between classes 1 and 2 are minor, they also belong to the same branch in the cladogram (Fig. 5). However, by looking at averages in the morphological data, it is clear that seeds in class 2 have relatively longer pips length (L), stalks (LS) and narrower breath (B) than class 1. The traits that most grape morphological specialists have ascribed as traditional domestication characteristics are more obvious in class 2, which might represent a more derived domestication stage.

Subsequent LDA results also confirmed the conclusion of a strong discrimination between all 4 classes. The model shows that 93.60% of the grape seeds have been correctly assigned, consistent with the results of UPGMA cluster analysis, proving the reliability of the UPGMA classification. However, according to the confusion matrix and classification results obtained by LDA, some specimens may be overlapping between classes 2 and 1 and class 4 and 3. The potential for overlap further attests to the similarity between these paired groups, which is also consistent with our statistical results in L, B, LS, and PCH. Also, taking the grape morphological groups found in archeological sites as a reference, using the DA method to classify modern cultivated grapes, we show that all of the modern local cultivated grapes that we collected are classified into class 2 (*p* value > 0.75), which further confirms that class 2 has obvious characteristic traits of modern domesticated forms.

### Grape morphological diversity and human selection in Bronze Age Central Asia

The domestication and dispersal of grapes remains a heavily debated topic. The wild grape (*Vitis vinifera* L. ssp. *sylvestris*), is dioecious and produces small, black berries, which naturally grows across the Mediterranean, east to the western slopes of the Himalayas, but the domesticated grape *(V. vinifera* L. ssp. *sativa*), is characterized by larger berries and hermaphroditism^27^. The earliest evidence of grape processing and utilization by humans can be traced back to 8000 BP in West Asia and the Caucasus, and scholars have argued that people were foraging and even fermenting grapes for many millennia prior to their cultivation^26,28,47–49^. Current research suggests that wild grapes produce roundish pips with short stalks, while domesticated seed varieties are more elongated with longer stalks. These differences have been acknowledged for a long time and widely used in archaeobotanical literature to discriminate wild and domesticated pips in archeological grape seeds^37,50^. Although, it is also well recognized that there is considerable overlap in these variables in early cultivated forms and all early cultivated forms are morphologically classified as wild and many domesticated populations contain individual seeds that would overlap with wild forms. Ultimately, discussions of morphometrics in grapes are only meaningful on a population level. Meanwhile, carbonization process also can changes the seed form, some researcher even questioned the reliability of distinguishing wild or cultivated varieties by measuring charred seeds^28^. However, according to recent experimental research, the deformation of the charring process does not greatly affect the result of morphological identification^51,52^. These realizations, combined with the traits that we just discussed, seems to suggest that there is variation in the archaeological specimens that we present here, with some having more wild affinities and others representing more derived forms.

In Transoxiana, wild grape relatives may have been present across western Turkmenistan and the Caspian Sea region in the past, but as of yet no grape seeds have been recovered from early archaeological village sites, such as Jeitun^53,54^, and they continue to remain absent in assemblages dating through the occupation at Sarazm (5500 cal. BP)^11,16,26^. There has been a considerable focus on the spread of crops across Eurasia and archaeobotanical inquiry over the past decade^55,56^, and, despite need for more early archaeobotanical assemblages from this region, it seems likely that the lack of earlier evidence is an indication of the fact that the rise of the Oxus city states marked the advent of viticulture in Central Asia. Grape cultivation had already made its way into the Indus region by 5000 BP, but does not really become prominent until the rise of the Indus Civilization around 4500 BP^57^.

The PCA and LDA results show that the grape seed morphology within cultivated populations dating to roughly 4000 BP in Central Asia can be effectively divided into 4 class. Among them, class 2 has the strongest affinity towards domesticated characteristics, but the quantity of seeds in this grouping is relatively low, accounting for only 15.2% of all seeds. Compared with the intermediate class 1 and the strongly domesticated class 2, the most marked characteristics of class 3 and 4 are shorter seed lengths and stalks, and the lengths of seeds in class 4 are shorter than those in class 3 and the breath is larger, features which are generally considered as indicators of wild grapes, accounting for 40% of all grape seeds in the study.

Most of the grape seeds found at Djarkutan and Sapalli belong to classes 1 and 3, accounting for 80% of the overall assemblage. Among them, class 1, which accounts for 45%, has the most similar morphology to class 2, with some derived characteristics. The cross-validation results and confidence ellipses between classes 1 and 2 in the LDA also show some overlap. All of these observations are consistent with previous research in Europe that found a large number of archaeological grape specimens that fall along a continuum between domesticated and wild groups from southern and western Europe^37^. Morphological studies of modern cultivate grape variation in Europe have shown that the elongation gradient of grape seed length and stalk length might be represented as a marked continuum of the domestication syndrome. Again, this leaves open the question of whether different forms were being selected and preferred, notably whether class 2 might have represented a more recently introduced and more derived form or an example of further localized parallel evolution of domestication traits. It also leaves open the question of whether class 1 represents a more ancestral form, possibly remaining in its ancient form due to continued asexual propagation. Also, we must admit that the pips deformation process during carbonization might affect our judgment on these issues.

### Grape seed morphology and early horticulture

In Central Asia, archaeological sites are disproportionately more prominent in areas where there is current human activity, notably in the piedmont oases, as the arid climate of the deserts and steppe makes agricultural pursuits more challenging^58^. Due to limited water availability, summer agriculture mainly relies on irrigation in these regions^59^. Fruit cultivation outside the foothills, notably in the arid desert, requires a significant investment in labor and time, arguably indicating the presence of local-scale Wittfoglian-style hydraulic city states and some form of centralized authority by this time. Although, small-scale garden-style grape cultivation is possible if farmers were familiar with the necessary soil conditions, drainage requirements, and need for water inputs in the summer^60^. Grape vines are also a delayed return crop, and when asexually reproduced they tend to require a minimum of four years before the first harvest is procured^49,61^. At the same time, early urbanization of southern Central Asia, economic development, and population increases would all have led to an increased demand for fruit, wine, and cash crops.

Archaeological and historical evidence attests to the economic and ritual significance of wine across Eurasia. Wine-related artifacts are readily found in tombs and ceremonial contexts, and are generally considered to have been used as mortuary offerings, during feasting events, and as expressions of conspicuous consumption in the past^62,63^ Historical sources attest to the cultural significance of wine across the Mediterranean and from most cultures of the ancient Silk Road^63,64^. Historically, grapes and wine making have been an important part of the Central Asian cultural repertoire, art historical depictions of grape vines are prominent across medieval sites and wine is readily attested from Sogdian and later sources^64,65^. The earliest archaeological evidence for wine production comes from the Lake Urmia basin in northwestern Iran and from roughly the same time period in the mountains of Georgia (8000 cal. BP), all of which is presumed to have relied on truly wild populations of grapes^26,48,66^. Clearer evidence for wine production, likely on a larger scale, dates to ca. 5000 BP in Anatolia^62^ and northern Greece^36,67,68^. Other research suggests that the earliest domesticated grapes were present in the Caucasus by around 6000 BP^69^ and the first cultivated morphotypes introduced to that region were still on a trajectory towards a more modern-looking domesticated form, some of these morphologically look more derived and others looking more plesiomorphic.

The deeper cultural legacy of prehistoric viticulture in southern Central Asia requires further investigation. Grape pips have been recovered 3500–3000 BP site across the Tien Shan and into Xinjiang, attesting to widespread cultivation of grapes in ecologically amicable regions by that time period^70,71^. Large-scale wine production is also attested in the earliest Chinese accounts of peoples in Central Asia (notably in a region that historians generally accept to be Ferghana)^72^ and the earliest Classical sources that discuss cultural attributes of peoples to the north (although the exact peoples discussed are less clear)^73^. The continuing importance of viticulture across southern Central Asia is attested by the discovery of an ancient winery at Kerkidon with two large vats, each having the capacity to hold 400 L, dating to 2200–1700 BP^74^. The best evidence that grape cultivators were selecting specific varieties and preferentially propagating favorable morphotypes comes from the ostraca at Nisa (2300–1600 BP.), which contain written references to different types of wine, classified by color and age, and noting if it had turned to vinegar^75^. The grape pips that we present in this paper are temporally on par with the oldest ever recovered in southern Central Asia. Ancient grape seeds have been previously reported at Anau South in Turkmenistan (4500 cal. BP)^76^, and both Gonur Depe and Djarkutan (4000 cal. BP)^26,77^. Artifacts that archaeologists ascribe to pottery wine vessels have also been found at early sites, such as Sarazm and Sapalli, but they require further investigation^10,78^.

**Figure 6:**
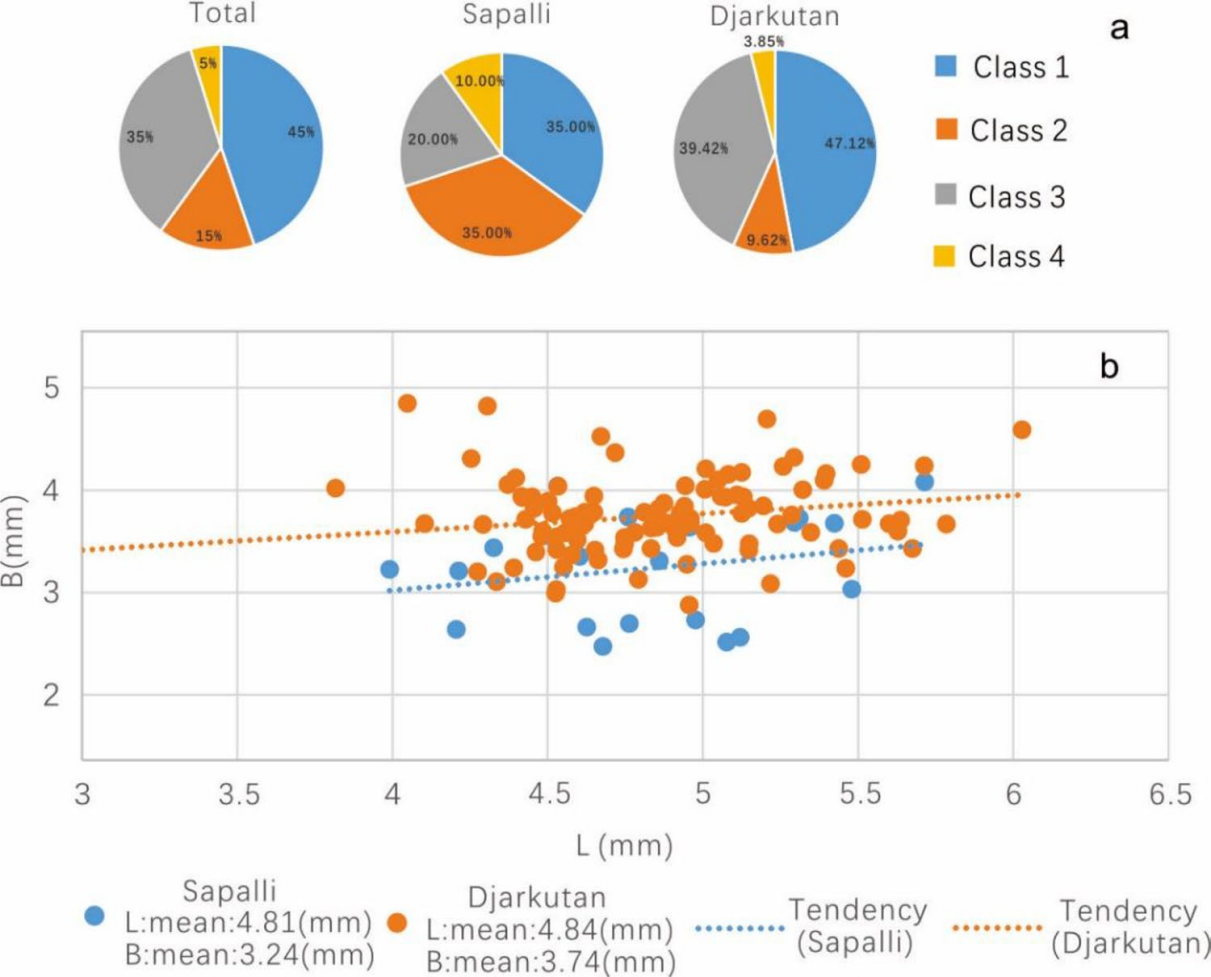
The assemblage and volume trends of grape seeds found in the two different sites, from Bronze Age Central Asia. (**a**) Distribution of grape pip classes in the Djarkutan temple and Sapalli. (**b**) Average volume trend of grapes seeds in the Djarkutan temple and Sapalli.

The grape seeds that we report here from Djarkutan come exclusively from a temple context, whereas those from Sapalli come from domestic fill, While only limited interpretation can be made based on the comparison of two data points (Fig. 6a), it is worth pointing out differences in the assemblages. In the Djarkutan assemblage, classes 1 and 3 specimens occupy about 80% of the total. Class 2, the form with a more domesticated affinity was rare in the Djarkutan assemblage, while the proportions of classes 1 and 2 were basically the same between the Sapalli and Djarkutan. Considering the small number of measurable grape pips from Sapalli, these details should not be over interpreted. However, the concentration of the more domesticated morphotype and large pips of grape in the temple may indicate locals will select some specific grape berry for ritual activities or for more elite contexts. A large number of clay pots were found under the room floor in the northwest corner of the temple^79^, possibly representing ritual offerings or consumption. By further comparing the grapes found in the temple with the grapes found in the domestic contexts, we note that those recovered from the temple are significantly larger (Fig. 6b).In modern wild grape research, small berries produce small roundish pips while large berries produce larger and elongated pips^37^. Consider about the large number of wild and intermediate compartments in Djarkutan, it is possible that these large pips grape represent a form that local people recognized to be better, due to the larger size, and therefore cloned it to propagate in the gardens or fields that produced for the temple. Alternatively, the fields that the grapes for the temple were harvested from may have prospered from greater irrigation and water input, possibly resulting in larger fruiting specimens. We also need to point out that there are some differences in the preservation of grape pips between two sites, pips found in Djarkutan were more severely charred than Sapalli. Modern carbonization experiments showed the morphological changes during the carbonization process are mainly increased in breadth and shortened in length^51^. While the average pips length in Djarkutan was not significantly shortened while the breadth was significantly increased than Sapalli. That indicates the morphological changes during the carbonization process do not affect the conclusion made above.

### Conclusions

Based on morphometric analyses, we classified the ancient grape seeds recovered from two Bronze Age urban contexts in Bactria into 4 classes, including 2 with affinities towards wild forms, an intermediate type between wild and domesticated, and a morphotype that more closely resembles modern domesticated types in the region today. Given the clonal nature of grapevine propagation, these morphotypes may represent different genets or closely related stages in a hybrid complex. The abundance of intermediate types was greater, while the number of more derived forms was less. Comparing the samples between the two sites in this study, while having obvious limitations, does open further questions about the likely recognition of specific morphotypes or genets that may have been asexually reproduced by farmers. Notably, the specimens from the temple context appear more likely to be both larger in overall size and also express features more closely aligned with modern domesticated forms. While we speculate that these phenotypic differences are genetic-based, we do not rule out the possibility that the larger forms are expressing at the extreme end of a plasticity reaction norm, i.e. they were receiving more water and nutrient inputs during seed development. With the early urban development, the increased demand for wine consumption from elites, mortuary and ritual activities may have increased demand for wine and grapes, the cultivation of which would have been tied to reliable irrigation systems. We also provide radiocarbon dates for these Bronze Age urban sites in Bactria, and a preliminary study of the local carbonized grape remains, providing important insights for further research on the domestication and diffusion process of grapes in the Old World.

## Methods

### Archeological sample collection

The flotation work was carried out at Sapalli and Djarkutan site after excavation during 2017 (Fig. S1 and S2). A total of 51 flotation samples were collected from the southeastern corner of Sapalli, which has been tentatively interpreted as a storage area. Temple samples from Djarkutan were collected from room 5 next to the main altar. Earlier studies of room 5 have interpreted this area as an ashes deposit for the main altar^79^, while we do not specifically defend this interpretation, we do agree that the burnt material is associated with the temple. A total of 3 flotation samples were collected in room 5, which include a large number of carbonized plant remains. Each flotation sample was more than 20 L and processed using a 0.3 mm mesh sieve. The material obtained by flotation was air-dried in the shade, relative analyze such as sorting, classification, identification, and photography were carried out on a Leica M205 C stereomicroscope and LAS V4.12 software in the Key Laboratory of Vertebrate Evolution and Human Origin of the Institute of Vertebrate Paleontology and Paleoanthropology, Chinese Academy of Sciences.

### Modern sample

Wild grape resources in Central Asia are restricted to the southern area of the Caspian Sea and far western of Turkmenistan, the eastern region has been considered to lay outside the tradition distribution area of wild grapes. To better understand the grape seeds morphology found at the archeological site, we collected the mean morphological data from about 25 western European cultivars and wilds recovered from previous studies as modern reference material^37^. Modern comparative material mainly recovered from France, utilization include two types of winemaking and fresh consumption.

### Chronology

Some seeds were selected from the carbonized plant remains for AMS^14^C dating, all samples were sent to the US BETA laboratory for testing. The pretreatment protocol followed the acid–alkali-acid method, the sample was first ground and dispersed in deionized water, then submerged in heated HCl to remove carbonate, following these steps, NaOH was used to remove organic acid. Finally, the sample was washed and neutralized with acid then dried. The ^14^C dates were subsequently calibrated using OxCal v 4.4 software and the IntCal20 database^80,81^.

### Image acquisition

A total of 125 grape seed remains were selected with complete shape, less deformation and less clear alteration during carbonization for further analyze. The grape seeds were subjected to 225 kV three-dimensional (3D) fossil micro-CT (225-3D-μCT) scanning (Developed by the Institute of High-Energy Physics, Chinese Academy of Sciences)^82^. Scanning parameters were set as follows: voltage 140 kV, current 120/100 μA, 360-degree rotation scan, step 0.5 degrees, 4 scans per angle, scan resolution 8.67 μm (X, Y, Z axis consistent). The raw projection was converted into a tomographic slice in .raw format by using IVPP225kVCT_Recon software, 2048 * 2048 pixels consisted each tomogram. The raw tomogram was imported into VGstudio 2.2 software and saved as a .Tiff file, then imported into Mimics 19.0 software to separate the pedestal and sample to obtain the 3D structure. Separation was performed by multiple modification and thresholding function tools in Mimics 19.0. Related measurements were also performed using Mimics 19.0.

### Statistical methods

According to previous research^37,50^, we selected 4 linear measurements, which are regarded as the most efficient to discriminate between wild and domesticated grapes and are easily observable, including total length (L), length of stalk (LS), position of the chalaza (PCH) and total width (B) (Fig. 2a). Also referring to previous studies^37^, in order to minimize the effect of size and focus on shape, discrete measurement data were transformed into log shape ratios of each variable divided by the geometric means of all 4 variables then logarithmically transformed^83,84^. Principal component analysis (PCA) was carry out by log-shape ratio to obtain grape seed shape variation. Cluster analysis was also performed using the unweighted pair-group method with arithmetic means (UPGMA) on the PCA centroid coordinates to explain the highest levels of morphological variation (PC1 and PC2). At the same time, linear discriminant analysis (LDA) was performed using log-shape ratios to test the validity of the subgroups identified by PCA and UPGMA cluster result. All statistical analyses were performed using XLSTAT 2019 (AddinSoft, Paris).

## Supplementary Information

Supplementary Information 1.

Supplementary Information 2.

Supplementary Information 3.
