# Supplementary material for: Morphotype broadening of the grapevine (Vitis vinifera L.) from Oxus civilization 4000 BP, Central Asia
Source: Sci Rep. 2022 Sep 29;12:16331. doi: 10.1038/s41598-022-19644-0 (PMC9522827; doi:10.1038/s41598-022-19644-0)
Supplement: Supplementary file 2 — Supplementary Information 2. [file 41598_2022_19644_MOESM2_ESM.docx]

**Supplementary material：**

**Research area and Archeological site：**Archaeological samples were collected from Sapalli and Djarkutan site in Surkhan Darya region of southeastern Uzbekistan. In the 1960s and 1970s, archaeologists in the former Soviet Union found a large number of settlements belonging to multiple periods in the area and carried out a great deal of archaeological work^1-3^. Rainfall in this area mainly focused on spring, vegetation is dominated by low shrubs and steppe^4,5^. Historically, the fertile valley has in this area have been an ideal place for the development of intensive agriculture, it is also the connection between the inner Asia mountain corridor and the northern steppe^6,7^.


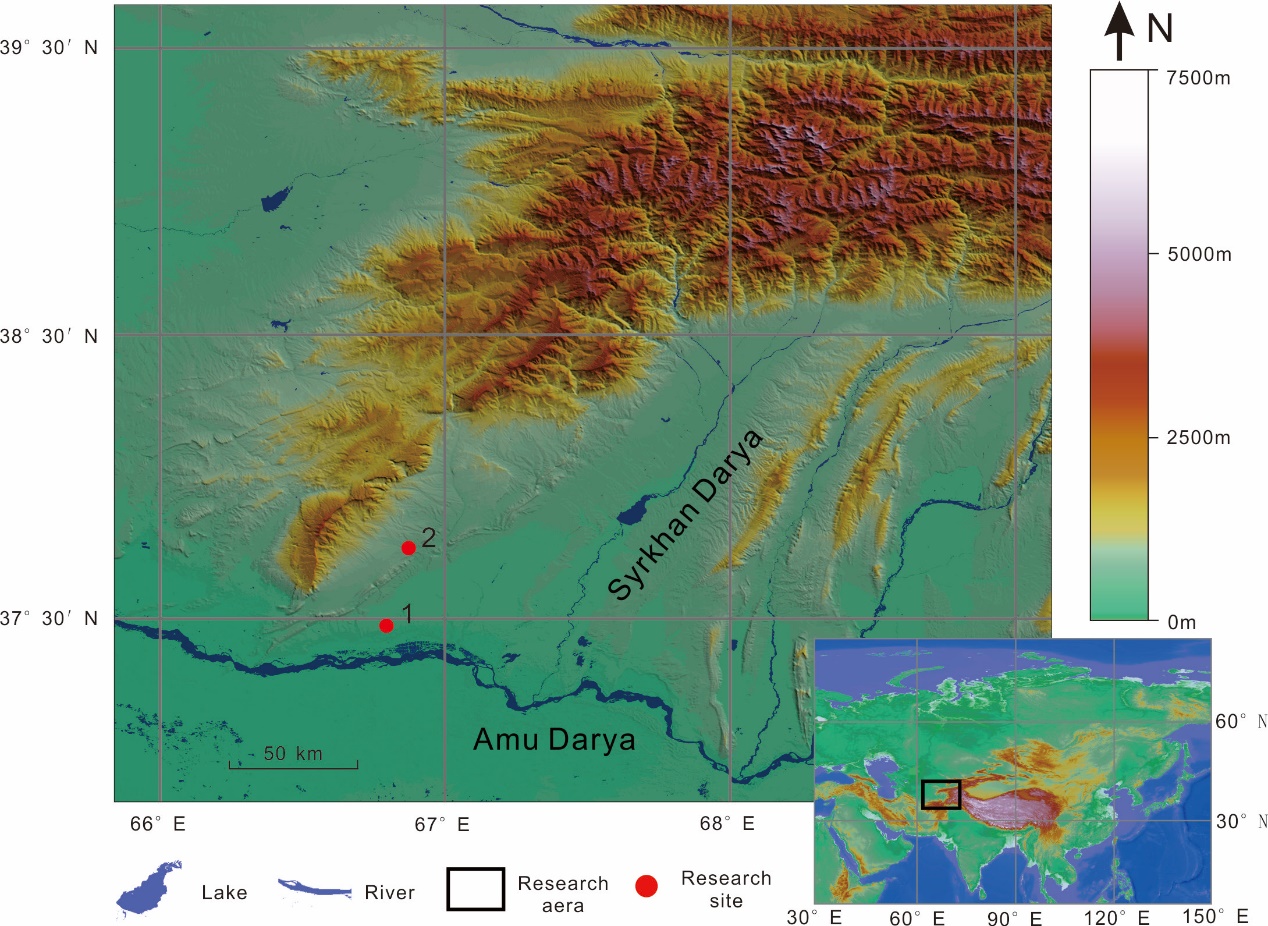


Figure S1 Location, topography and site distribution of the study area. 1-Sapalli；2-Djarkutan. This maps were created using ArcGIS v10.6 (https://www.esri.com/), in-map labels were added in CorelDraw X8 v18.1.0.690 (https://www.coreldraw.com/).

Sapalli tepe is the most representative site of the earliest agricultural civilization in Bactria region. The Sapalli culture, named by this site, represents the late Bronze Age settled agricultural culture in the Uzbekistan and southern Tajikistan, which can be divided into 4 stages, include Sapalli; Djarkutan; Kuzalinksi and Molalli^2,8^. The main structure of this site is a square farm or fortress (Fig. S2a), with a side length of 82 meters, surrounded by mud brick wall and rectangular corridor-shaped rooms, which probably the false entrances. In the center of this site is a square, surrounded by residential areas build in three periods. 138 graves were also uncovered during the excavations, many of them were situated under the floors of the residential or streets, surrounding by rooms and corridors, which include many materials like ceramics, metal object, jewelry, and some ritual objects like seal-emblems.


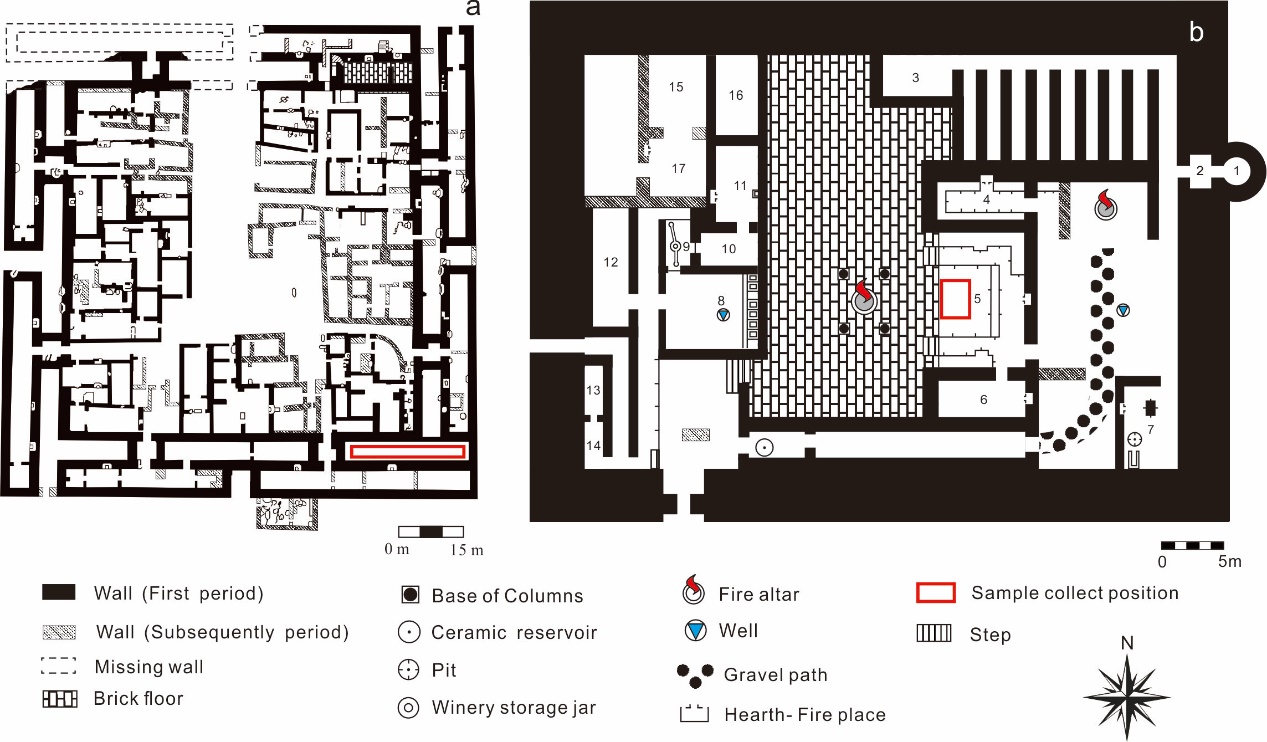


Figure S2 Plan and sample collection position about archeological site. a Sapalli；b Djarkutan

The Djarkutan site, located 60 km north of Termez city, is one of the largest Bronze Age urban site in Bactria region and the center of the Sapalli-Dashli culture^2,3^. This site covers more than 100 hectares and no obvious city wall around. The boundaries can be clearly distinguished by hills and trench in north and west, east part is gradually flat without obvious boundaries, south part is covered by a public cemetery, separated by a canal with the settlements on the north side. From south to north respectively appear cemeteries, canals, residential areas, temples and Palace^9^.

The temple (Figure S2b) is located on the east part, 300m south of the castle and 50m from the residential area, might be used as the public religious activity center in this site. The architectural of this temple is typical Sapalli cultural form, including surrounding corridors, monolithic encircling wall and complexes of outbuildings, which include 3 buliding perioud, corresponding to Djarkutan, Kuzalinksi and Molali period in Sapalli culture^9,10^.

| Lab NO. | Sample NO. | Marital | Age / BP | σ | Calibrate age / Cal. BP |
| --- | --- | --- | --- | --- | --- |
| Beta-459964 | SPL-1 | wheat | 3540±30 | 2 | 3908-3700 |
| Beta-637137 | SPA 3-2 | Grape | 3500±30 | 2 | 3869-3650 |
| Beta-527178 | Djarkutan T1 | Grape | 3560±30 | 2 | 3969-3723 |
| Beta-527179 | Djarkutan T2 | Grape | 3580±30 | 2 | 3977-3731 |
| Beta-527180 | Djarkutan T3 | Grape | 3520±30 | 2 | 3880-3698 |

Table S1 AMS^14^C dating results of Sapalli and Djarkutan temples

1 Childe, V. G. The urban revolution. *Town. Plan. Rev.* **21**, 3-17 (1950).

2 Askarov, A. Southern Uzbekistan in the Second Millennium DC. *Sov. Anthropol. Archeol.* **19**, 256-272 (1981).

3 Dani, A. H. & Masson, V. *History of Civilizations of Central Asia Vol. I The Dawn of Civilization: Earliest Times to 700 BC*. (UNESCO Press, 1992).

4 Sataev, R. & Sataeva, L. in *Proceedings of the 8th International Congress on the Archaeology of the Ancient Near East. ICAANE 8.* 367-370.

5 Egamberdieva, D. & Öztürk, M. *Vegetation of Central Asia and Environs*. (Springer, 2018).

6 Lerner, J. Regional study: Baktria–the crossroads of ancient Eurasia. *The Cambridge World History: Volume 4, A World with States, Empires and Networks 1200 BCE–900 CE* (2015).

7 Holdich, T. H. *The gates of India: Being an historical narrative*. (Good Press, 2019).

8 Пугаченкова, Г. А. *Халчаян* 1-287 (Наука УзССР, 1966).

9 Askarov, A. & Shirinov, T. The" Palace," Temple, and Necropolis of Jarkutan. *BAINS* **8**, 13-25 (1994).

10 Djuraeva, S. Zoraastrism and Zoolatric views at the mounment Jarqutan which situates at the south Uzbekistan. *Theor. Appl. Sci.*, 166-168 (2019).
